# Plants as Useful Vectors to Reduce Environmental Toxic Arsenic Content

**DOI:** 10.1155/2014/921581

**Published:** 2014-01-09

**Authors:** Nosheen Mirza, Qaisar Mahmood, Mohammad Maroof Shah, Arshid Pervez, Sikander Sultan

**Affiliations:** ^1^Department of Environmental Sciences, COMSATS Institute of Information Technology, Abbottabad 22060, Pakistan; ^2^Department of Microbiology and Molecular Genetics, University of the Punjab, Quaid-i-Azam Campus, Lahore 54590, Pakistan

## Abstract

Arsenic (As) toxicity in soil and water is an increasing menace around the globe. Its concentration both in soil and environment is due to natural and anthropogenic activities. Rising arsenic concentrations in groundwater is alarming due to the health risks to plants, animals, and human beings. Anthropogenic As contamination of soil may result from mining, milling, and smelting of copper, lead, zinc sulfide ores, hide tanning waste, dyes, chemical weapons, electroplating, gas exhaust, application of municipal sludge on land, combustion of fossil fuels, As additives to livestock feed, coal fly ash, and use of arsenical pesticides in agricultural sector. Phytoremediation can be viewed as biological, solar-driven, pump-and-treat system with an extensive, self-extending uptake network (the root system) that enhances the natural ecosystems for subsequent productive use. The present review presents recent scientific developments regarding phytoremediation of arsenic contaminated environments and its possible detoxification mechanisms in plants.

## 1. Introduction

Arsenic is a trace metalloid found in almost all environments. It exists in the −3, 0, +3, and +5 oxidation states. Environmental forms include arsenious acids, arsenic acids, arsenites, arsenates, methylarsenic acid (MAA), dimethylarsinic acid (DMAA), trimethyl arsine oxide (TMAO), and so forth [1–3]. The most abundant forms of arsenic include arsenate (As V) and arsenite (As III) [4], where arsenite is a toxic and hard acid. Arsenate (As V) usually forms complexes with sulfides, whereas arsenite (As III) develops complexes with oxides and nitrogen chemical species. Under both the oxidized and reduced states, As is sensitive to mobilization at pH range of 6.5~8.5 [5].

Arsenic toxicity of soil and water is an increasing menace across the globe [5, 6]. Millions of people especially in developing countries of Southeast Asia and many other regions are chronically exposed to As contamination [7, 8]. As carcinogenicity is well documented as it seriously affects human health and causes bladder, lung, and skin cancers and possibly damage to liver and kidney as well [7, 8]. Noncancerous health effects of As exposure include diabetes, skin diseases, chronic cough, and toxic effects on liver, kidney, cardiovascular, and nervous system [7–11]. Moreover, As contamination has become a major environmental concern because it not only adversely affects humans but also causes highly toxic effects on metabolic processes of plants, mitotic abnormalities, leaf chlorosis, growth inhibition, reduced photosynthesis, DNA replication, and inhibition or activating enzymatic activities [12].

## 2. Arsenic Concentrations in Soil and Water

Arsenic is the 20th most abundant element in earth crust [13], making about 5 mg kg^−1^ of earth's crust, with an average concentration of 2 mg kg^−1^ in igneous and sedimentary rocks [14]. It is a naturally occurring element typically found in soil at background concentrations ranging from 0.1 to 40 mg kg^−1^. As is commonly associated with sulfides, oxides/hydroxides of aluminum (Al), iron (Fe), and manganese (Mn); other sources are volcanic eruptions and sea salt sprays [15]. In soil, As is present in the form of oxides, hydroxide, chlorides, and sulfides, such as enargite (Cu_3_AsA_4_), cobaltite (CoAsS), and skutterudite (CoAsS_4_) and its average concentration in different regions of the world is 9.36 mg kg^−1^. Heavy use of As containing pesticides is considered as the major reason for its pollution [16]. Arsenic and P are chemically similar. Both form insoluble compounds with Al and Fe in soils. In soil, Al-As and Fe-As complexes are the dominant chemical forms, while arsenic has less affinity for Al oxides than phosphates. As (III) gets adsorbed on iron (III) surfaces [17]. Kaolinite and montmorillonite have higher affinities for As (V) than for As (III) [18]. Arsenic mobility and phytotoxicity are greater in sandy soils.

Rising arsenic concentrations in groundwater are alarming due to the health risk to plants, animals, and humans health [19]. Higher levels of arsenic were found in groundwater sources than in surface-water sources. Many countries around the world (including Taiwan, Argentina, India, Bangladesh, Mexico, Hungary, and Chile) have reported extensive arsenic groundwater contamination [19, 20]. Use of such contaminated water for irrigation of crops may lead to arsenic contamination of agricultural soils. The presence of high As concentration in the aquifer may be due to desorption of arsenic from Fe and Mn oxides, weathering of primary silicate minerals, and apatite under high pH and alkalinity from silicate and carbonate reactions [21].

### 2.1. Anthropogenic Sources of Arsenic

Anthropogenic As contamination of soil may result from mining, milling, and smelting of copper, lead, and zinc sulfide ores, hide tanning waste, dyes, chemical weapons, electroplating, gas exhaust, municipal sludge of land, combustion of fossil fuels, As additives to livestock feed, coal fly ash, and agricultural use of arsenical pesticides [3, 22–30]. In the past decade, the global input of As to soils by human activities was estimated to be around 52,000–112,000 ton per year [31]. Thus, the arsenic concentrations in soil and environment both are due to natural and anthropogenic activities. Most of the arsenic risk is associated with the forms that are biologically available for absorption or “bioavailable” to plants and humans. A bioavailable chemical is the portion of a chemical dose that enters the systemic circulation from an administered dose [32].

Of 1.4 million worldwide contaminated sites 41% are in the USA and US EPA has recognized that arsenic (As) concentration in Australia was greater than 10,000 mg kg^−1^ [33]. Arsenic has been found at high levels (10 000–20 000 mg kg^−1^) in some contaminated areas and that results in unacceptable levels of risk to human health from the incidental ingestion of soil [34]. Groundwater arsenic contamination has been reported in many parts of the world, such as Vietnam, Massachusetts State, Carolina State, Canada, and Bangladesh, with 0.305, 30, 2460, 6590, and 0.3990 mg kg^−1^ arsenic (As) contamination [35–40]. As intake through drinking water is a very severe problem in the Southeast Asia with the Bengal delta being the worst affected area [41]. Mining has resulted in increased As concentrations in Warsak Canal [42]. Large arsenic concentrations such as 0.942, 0.40, 0.38, 0.643, and 0.475 mg L^−1^ were found in Hattar Industrial Estates, Ghari Rahimabad, Pakha Ghulam, Peshawar Industrial Estate, and Gujranwala Industrial Estate, respectively, in Pakistan [43].

One of the more widespread problems is the leaching of naturally occurring arsenic into drinking water aquifers [3]. Thus, groundwater As contamination is the most common result of its higher concentrations in soil. It is estimated that approximately one third of the world's population use groundwater for drinking [44, 45], which ultimately adversely affects human beings as the biggest calamity, was in Bangladesh, where millions of people were dependent on As contaminated drinking water [12] and it is the possible cause of the death of such notables as Napoleon and the American president Zachary Taylor [3].

The reduction of the World Health Organization (WHO) provisional guideline value for As concentration in drinking water was from 50 *μ*g L^−1^ to a provisional 10 *μ*g L^−1^ in 1993 [46]. However, only during the past 5 years many industrial countries adopted that lowered guideline value as the maximum contaminant level (MCL). On the other hand many developing countries including India and Bangladesh still have 50 *μ*g L^−1^ as MCL [41] and the reduction of the maximum admissible concentration (MAC) to 10 *μ*g L^−1^ by USEPA in 2002 was a response to growing concern over that poisonous carcinogen which raised awareness of the dangers of As in drinking water [47]. In view of the health concerns outlined above, and alerted by the magnitude of the problem afflicting nearby Bangladesh and West Bengal, the Public Health Engineering Department (PHED), the Local Government and Rural Development Department (LGRDD) of Pakistan, in conjunction with UNICEF, undertook a survey of As concentration in groundwater from drinking water supply wells in Pakistan [48]. That survey revealed hot spots of As enrichment in parts of the Indus alluvial basin. The survey identified Muzaffargarh District (Pakistan) as one enriched in As at concentrations in the low hundreds of *μ*g L^−1^ range. During the investigation, the authors found As “cold spots;” that is, areas where evaporative concentration of groundwater might have been expected to result in high concentrations of As in groundwater, but where concentrations were, in fact, below levels of concern [47].

Soil contamination and groundwater can be due to industrial point sources, repeated use of metal enriched fertilizers, farm manuring, sewage sludge, pesticides application, mining, automotive emissions, dyestuffs, and wood preservation [49]. As arsenic concentrations above acceptable standards have been detected in many countries such as Bangladesh, Cambodia, China, Taiwan, Inner Mangolia, India, Iran, Japan, Nepal, Pakistan, Thailand, Vietnam, Alaska, Argentina, Chile, Mexico, United States of America, Austria, Finland, France, Germany, Greece, Italy, Russia, United Kingdom, South Africa, Australia, and New Zealand [50]. In some areas of the Pakistan, the presence of arsenic in subsurface aquifers and drinking water systems is a potentially serious human health hazard. A majority of shallow subsurface aquifers and tube wells are contaminated with arsenic at levels which are above the recommended arsenic level of 10 ppb.

Soil and water contamination can be removed by immobilization, vitrification, soil washing/flushing, precipitation, membrane filtration, adsorption, ion exchange, permeable reactive barriers biological treatment, thermal processes, excavation and disposal process, chemical processes, and phytoremediation costing 75–425, 100–500, 100–500, and 5–40 dollars per ton of soil, respectively [51]. Most of these methods are found very costly, whereas phytoremediation has been suggested as the most cost effective and efficient method for removal or minimization of metal contamination both in soil and water [52]. Phytoremediation was firstly proposed over 20 years ago and is advantageous over chemical stabilization, which may prevent health threats occurring due to leakage of toxic metals [53]. Phytoremediation has also been called green remediation, botanoremediation, agroremediation, and vegetative remediation. It is a natural process of growing plants to remediate soil and water without affecting the landscape. Phytoremediation utilizes biological processes and anatomy and physiology of plants. It is plant-based soil remediation system can be viewed as biological, solar-driven, pump-and-treat systems with an extensive, self-extending uptake network (the root system) that enhances the below-ground ecosystem for subsequent productive use [54, 55]. Phytoremediation is a continuum of processes occurring to varying degrees under different conditions, media, contaminants, and plants [54]. Plants have both constitutive (present in most phenotypes) and adaptive mechanisms (present only in tolerant types) to cope with the elevated metal concentrations [51, 56]. They can absorb and accumulate metals much higher than they need. The metals are generally accumulated in their aerial tissues [57].

## 3. Role of Phytoremediation

Numerous terms are being used simultaneously in the literature to refer to these processes and may overlap to some extent. Phytoremediation consists of four to five different technologies [54, 55], each having a different mechanism such as the following.

1. Phytoextraction or phytomining or phytoaccumulation: plants take up and translocate metal contaminants from soil to the above ground portions, which then are harvested to remove the contaminant from the site.
2. Phytodegradation or phytotransformation: plants disintegrate pollutants which may occur within the plant by the metabolic activity or breakdown of the pollutant external to the plant contributed by various organic compounds released into the rhizosphere.
3. Rhizofiltration: plants get rid of contaminants present in solution surrounding the root zone by adsorption or precipitation onto their roots or absorption of contaminants into their roots from the solution. This technique is used to clean contaminated water such as groundwater or a waste stream.
4. Phytostabilization: plants immobilize contaminants in the soil and groundwater through absorption and accumulation by root or precipitation within the rhizosphere.
5. Phytovolatilization: plants volatilize pollutants; they take up the pollutants from the soil or water in the transpiration stream and volatilize into the atmosphere in a modified or unmodified form.

Arsenic phytoremediation involves immobilization, fixation, and removal either as fixed in soil or accumulated in plant parts.

### 3.1. Role of Plants in Remediation of Arsenic

Plants require an adequate supply of all nutrients, as part of normal growth and development [58], including arsenic, for their normal physiological and biological functions. Deficiency of specific nutrient occurs when plants cannot obtain sufficient amount as required, whereas excessive supply of the same, through contaminated soil results in toxicity to plants. Recommended soil application by US EPA for arsenic (As) is 41 mg kg^−1^, whereas recommended standards by WHO for drinking water and effluents to be released by industries are 1, 0.01, and < 0.01 mg L^−1^. The global input of arsenic to soils by humans in the last decade was estimated between 52,000 and 112,000 t year^−1^ [31]. Arsenic contaminated sites can be remediated by utilizing the *ex situ *physical and chemical techniques [51]. But physicochemical remedies render the land futile for further use, during the process of decontamination, since they abolish all biological activities contributed by beneficial microorganisms, which are necessary for plant growth and development. Consequently, the ecosystems deteriorate with a decline in biodiversity. Arsenic contaminated sites usually have adverse soil conditions, that is, poor soil structure, low organic content, inadequate N and P, and so forth, and plants need to adapt to these hostile soil conditions as well as to the metal contamination.

Generally, prior to imposed selection, a species must be able to thrive and survive in As contaminated soil and/or water, for which it must possess appropriate variances [59]. Thus, only plants possessing tolerance show some preadaptation to these harsh conditions. Notable examples of such plants are *Andropogon scoparius*, ribwort plantain (*Plantago lanceolata *L., *Holcus lanatus*),mosses, lichens, crowberry (*Empetrum nigrum *L.), Tamarix (*Tamarix parviflora*), Eucalyptus (*Eucalyptus camaldulensis*), and Chinese Brake fern (*Pteris vittata *L.) [59–68]. Tolerance of plants to metals is under control of uptake systems which are directly related to metal concentrations in the soil solutions. Plants mostly possess two uptake systems: the highly inducible high-affinity system operational at low concentrations (such as the high affinity phosphate uptake system under low phosphate status) and the constitutive low-affinity system that is effective at high concentrations [51, 61, 69–71]. For uptake, arsenic needs to be bioavailable. Two mechanisms are responsible for arsenic transport from the bulk soil to plant roots, mass flow, and diffusion. Thereafter, plants may utilize two separate systems to take up arsenic: (1) passive uptake through the apoplast and (2) active uptake through the symplast [51]. Once arsenic is taken up, it is translocated from the roots to the shoot system via the xylem and redistributed between tissues. The translocation of arsenic and other metals depends upon root pressure and leaf transpiration [51, 72]. Most plants take up arsenic as arsenate [73] since arsenite is unstable as it gets oxidized to arsenate by biochemical processes in the soil system. Arsenate being a chemical analogue of phosphate competes with phosphate for its uptake system and is actively taken up [61, 74, 75]. Once taken up, it is reduced in the cytosol to arsenite by glutathione (GSH) [76] and translocated to the shoots [77, 78].

Generally, only a minuscule amount of arsenic is translocated to the aboveground parts leading to little accumulation. The form in which arsenic translocated in plants was unknown until 1999 [79]. There was some evidence that arsenic transported as dimethylarsenic acid to the shoots [80] and may be stored as an arsenite-tris-thiolate complex [81] in tissues [82].

### 3.2. Detoxification Mechanisms in Plants

Large green plants have the capability to move large amounts of soil solution into the plant body through the roots and evaporate this water out of the leaves as pure water vapour during transpiration. Plants transpire water to move nutrients from the soil solution to leaves and stems, where photosynthesis occurs, and to cool the plant. During this process, contaminants present in the soil water are also taken up and sequestered, metabolized, or vaporized out of the leaves along with the transpired water.

Heavy metals are generally transported and deposited in the vacuole as metal chelates. According to Baker et al. [83], free metal ions in the solution are taken up by plants into their tissues and are reduced as metal chelates using specific high-affinity ligands (like oxygen-donor ligands, sulfur-donor ligands, and nitrogen-donor ligands), for example, carboxylic acid anions which are abundant in the cells of terrestrial plants and form complexes with divalent and trivalent metal ions of reasonably high stability. Carboxylates (such as malate, aconitate, malonate, oxalate, tartrate, citrate, and isocitrate) are commonly the major charge-balancing anion present in the cell vacuoles of photosynthetic tissues and several of these carboxylates get associated with high metal concentrations in plants [84–86].

Sulfur-donor ligands (like metallothioneins and phytochelatins) form highly stable complexes with heavy metals because sulfur is a better electron donor than oxygen. Metallothioneins are gene-encoded low-molecular-weight, cysteine-rich peptides found in fungi and mammals recently shown to be induced by Cu [87]. In fungi and mammals, metallothioneins are involved in metal detoxification [88] but their role in plants is not yet well understood.

**Figure 1:**
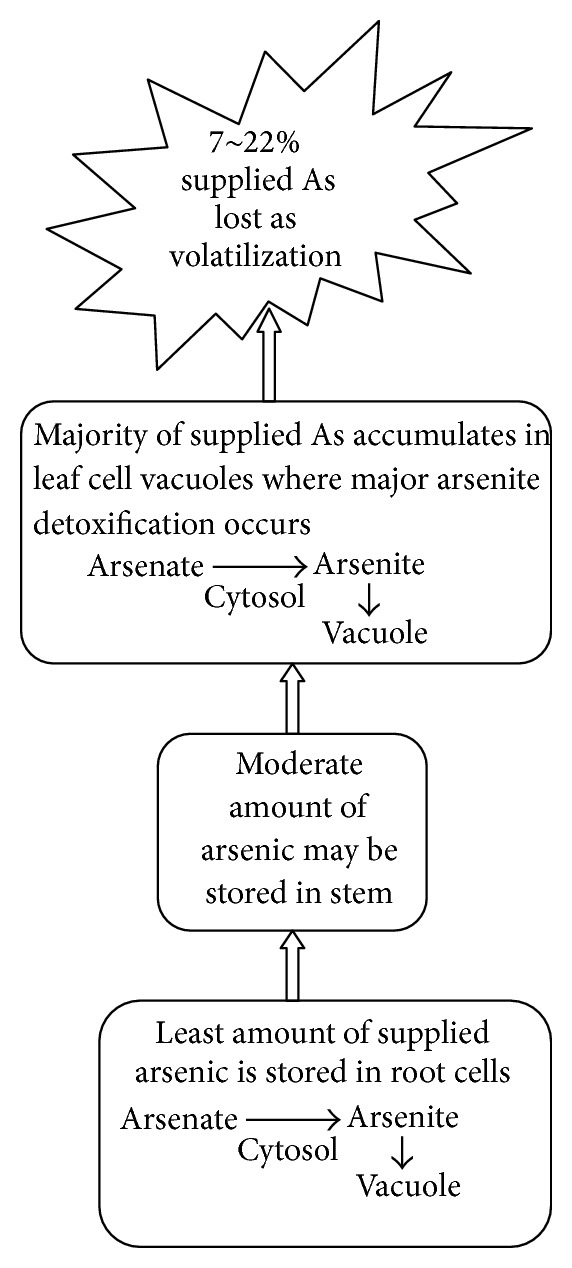
The overview of arsenic accumulation during phytoremediation experiments as suggested by the recent studies of Mirza et al. [91] and Doucleff and Terry [92]. It was suggested that arsenic is absorbed as arsenate and some part of it is converted into arsenite by an enzyme called arsenate reductase. Arsenite is stored in vacuole and is further detoxified. Majority of arsenate is transported to leaf cells where it is again converted into arsenite (in cytosol) and stored/detoxified in vacuoles. Still majority of investigators have proposed that arsenite form is transported from roots to shoots.

Plants employ several extracellular and intracellular mechanisms to detoxify heavy metals [51, 89]. These mechanisms include chelation, compartmentalization, biotransformation, and cellular repair [90]. The external mechanisms include exudations which change rhizosphere pH, metal speciation, and binds metal ions on the cell walls. Intracellular mechanisms include alteration of cell membrane or other structural protein to reduce the effects of metal toxicity and ultimate transport of metal to vacuole where detoxification occurs. Detoxification at the cellular level involves subcellular compartmentalization, chelation of metal in the cytosol by high affinity ligands, or binding metals to cell walls.  Figure 1 shows the possible As accumulation and volatilization in* Arundo donax* L.

For many contaminants, passive uptake via micropores in the root cell walls may be a major route into the root, where sequestration or degradation occurs. The apoplast is a hydrated free space continuum between the external soil solution and the cell membranes of the root cortex and vascular tissue. The cell wall micropores exist within a network of cellulose, hemicelluloses, pectins, and glycoprotein containing many negative charges (generated by carboxylic groups) that act as cation binding sites and exchangers and as anion repellers. Di- and polyvalent cations (the form of many heavy metal and radionuclide contaminants) are preferentially attracted to, and bound on, these cation exchange sites within the root cortex cell walls. For metal ions to be metabolized or translocated to the aboveground parts of the plant, they must pass through the plasma membrane of a living cell, and this can only occur by active transport processes. The inner limit of the root cell wall is the endodermis, which forms the outer limit of the root vascular system or stele.

Phytochelatins are low-molecular-weight, cysteine-rich peptides that are especially produced by plants when exposed to heavy metals and are known to bind metal in plants [93]. The PC-metal complexes are less toxic than free metal ions to cellular plant metabolism. Phytochelatin synthesis has been induced on exposure to arsenate in a number of plant species [79, 94, 95]. Intact PCs-As complexes have also been isolated from plant tissues [94] suggesting that phytochelatins are also involved in arsenic detoxification in plants. Though phytochelatin (PC) synthesis was induced on exposure to arsenate in *P. vittata*, only PC2 was detected in the plant. The molar ratio of PC-SH to As suggested that only a small proportion (1–3%) of the As in *P. vittata* can be complexed with PCs [95].

The metal-binding peptides, such as thiol-rich phytochelatins, have been most widely found and studied in plants particularly in response to arsenic [96]. They provide a detoxification mechanism by arsenic to their thiol group [79, 82, 94]. Where phytochelatins are derived from glutathione (GSH) [97] their biosynthesis is from GSH due to the presence of phytochelatin synthase enzyme [98]. Arsenic detoxification by phytochelatins on exposure to arsenate firstly was suggested by Grill et al. [99]. As it is understood that the immobilized metals are less toxic than the free ions, thus binding of arsenic to phytochelatins is considered to be a part of the detoxifying mechanisms of higher plants. After complexion; that is, the phytochelatin-metal complexes, for example, Cd-phytochelatin complexes, are transported to the vacuole which may be the final storage compartment where they either dissociate, degrade, or are shuttled back into the cytoplasm due to the acidic vacuolar pH [82, 100, 101]. On the contrary, if arsenic-phytochelatin complexes are transported inside vacuole, they might remain stable and prevent re-oxidation of arsenite due to the acidic pH of the vacuole, allowing accumulation of high concentrations of arsenic phytochelatin complexes [82].

Chintakovid et al. [102] pointed out that plants can tolerate the toxicity of arsenic by inhibiting translocation to the shoots, thus accumulating it primarily in the roots. Chintakovid et al. [102] found higher arsenic concentration in roots than in shoots of cotton exposed for a short time to arsenic. Although the mechanism of arsenic accumulation in the stem is still unclear, the results suggested that arsenic was transported to the stems and the leaves of the nugget marigold via the vascular system. The samples showed high percentages of arsenite in stems and leaves while a high percentage of arsenate was found in the roots. Similarly, the relative distribution of As in plants shows that *Brassica* sp. accumulated As mainly in the roots followed by shoots and flower [102].

It was found that arsenite was the main arsenic species in the fronds. Both species of arsenite and arsenate were found in xylem sap from stems of *Brassica juncea* [81] and sunflower [103]. However, it was not known whether both species were actually loaded in the xylem sap or occurred as a result of the reduction and oxidation of As species during translocation in the xylem sap. Raab et al. [103] found arsenic-phytochelatin complexes in the roots, stems, and leaves of an arsenic nontolerant plant (*Helianthus annuus*) during the exposure to arsenite or arsenate. But in most cases, most of the arsenic (75–95%) in the fronds is present in the form of arsenite (3+ oxidation state) [68, 104, 105]. Arsenite was also the predominant form of arsenic in excised aerial tissues that was exposed to arsenic, whereas As (V) was the main form in excised roots suggesting that As (V) reduction occurred mostly in the fronds, mainly in the pinnae.

### 3.3. Detoxification Mechanisms in *Arundo donax *L. 

*A. donax* is an erect, perennial, bamboo-like grass which has been present in the Mediterranean basin for thousands of years [106–108].* A. donax* has become globally dispersed by humans, so it is possible to find it in Asia, south Europe, North Africa, the Middle East, and also in North and South America and Australasia [106–108]. *A. donax* can be used for many purposes in the rural world, such as lattices, fences, baskets, fishing rods, and stalks for plants, roofs, windbreaks, sun shelters, cereal bins, musical instruments, walking sticks, and trellises. It is the most widespread among the species of the genus *Arundo. *It belongs to the Poaceae family of the Arundina tribe. The genus includes also *Arundo plinii*, *Arundo collina* and *Arundo mediterranea*.

It is considered one of the largest herbaceous grasses as its height could reach more than 8 metres [77, 108–110]. Several stems grow from the rhizome buds during all the vegetative season, forming dense clumps. *A. donax* stem is a hollow, segmented culm that measures from 1 to 4 centimeters in diameter and is able to branch during the second year of growth. Alternate leaves (5–8 cm wide and 30–70 cm long) are produced from the stem nodes, to which they are firmly wrapped [108, 111]. Stems and leaves are characterized by a relatively high content of silica, caused by the presence of siliceous cells associated with vascular bundles in the epidermal layer [108]. *A. donax* is reported to be a sterile species, and the propagation of this species is by agamic reproduction, occurring through regrowth of rhizome fragments and growth of shoots from stem nodes [112–114]. The adaptability to extreme soil conditions combined with rapid and vigorous growth makes *A. donax* an interesting subject for environmental studies on phytoremediation treatments. The use of plants to remove contaminants from polluted water and soil can be an advantageous strategy, which can also be used to remove metals that usually cannot be efficiently biodegraded. Studies indicate that *A. donax* may have a potential use for phytoremediation purposes. The plant is able to efficiently transfer arsenic absorbed from the growing medium and efficiently accumulate it into the shoots, showing a good tolerance to the presence of the metal [77].

Plant uptake and metabolism of arsenic has recently been reviewed by Tripathi et al. [115] and Zhao et al. [116]. Arsenic in the environment mainly exists in two inorganic oxidation states, arsenate (As (V)) and arsenite (As (III)). Phytoremediation of metals is the ability of plants to continually accumulate and detoxify metals in their system. In soil, arsenic exists in two forms, arsenate, thermodynamically stable under aerobic conditions, and arsenite under anaerobic conditions. As (V) is believed to interfere with oxidative phosphorylation, while As (III) may inhibit enzymatic activity by binding to thiol group. As (V) and As (III) enter plant cells via phosphate transporters and aqua glycophorins, respectively, as reviewed by Bhattacharjee et al. [117].

Once taken up, As (V) is reduced to As (III), catalyzed largely by arsenate reductases, members of the super family of protein tyrosine phosphatase (PTPase) [118] as shown in Figure 1. As (III) can then be complexed with glutathione (GSH) or phytochelatins (PCs). Raab et al. [103] identified up to 14 different species of arsenic complexes in sunflower plants. As (III) or complexed As (III) is then transported across the tonoplast and sequestered in the vacuole. Most data support the idea that arsenic is translocated from the roots to the tissues above ground, mostly in the form of As (III) [119, 120]. As (III) can be methylated to form monomethyl arsenate (MMAs (V)), dimethyl arsenate (DMAs (V)), and trimethyl arsine oxide (TMAO (V)) in plants [116, 121]. Complexation of As (III) with PCs or GSH is an efficient way to detoxify arsenic, probably because the complexes are pumped and sequestered in the vacuole catalyzed by the homologues of multidrug resistance proteins (MRPs), members of the ABC super family [122].

Although these studies indicated the feasibility of over expressing phytochelator synthase (PCS) and/or *γ*-glutamylcysteine synthetase (*γ*-ECS) for increasing arsenic accumulation and concomitantly tolerance, there are no direct data on the site of arsenic storage in these transgenic lines; thus it remains unclear whether the complexed As (III) is primarily vacuolar or remains in the cytoplasm. It is possible that transport of complexed As (III) or even free As (III) across the tonoplast membrane is a potentially the rate-limiting step in overall arsenic tolerance and accumulation. Yet, to date, there are no reports of genetic engineering of tonoplast transport [123].

One of the key properties of arsenic hyperaccumulators such as *Pteris vittata* is a highly efficient system of arsenic translocation from root to shoot [119, 124], while most non-hyperaccumulators usually have a low mobility rate compared to *P. vittata.* Arsenic mobility from root to shoot varies considerably among different plant species, suggesting that it is under genetic control. A key step in arsenic translocation from root to shoot is arsenic loading to the xylem, a process that is not well understood. Ma et al. [125, 126] identified a gene encoding an efflux protein, Lsi2, which is responsible for arsenite loading into the xylem, as arsenite is the dominant arsenic species in the xylem. An Lsi2 mutation resulted in a nearly 50% reduction in arsenic accumulation in the shoot. Lsi2 is a homologue of the *E. coli* ArsB, which is an As (III)/H^+^ exchanger that confers bacterial arsenite resistance [127]. The plant efflux protein apparently transports both metalloids As (III) and Si (IV). Methylated arsenic species have been detected in several plant species, including rice grain [128, 129], and recent data suggest that this is the result of endogenous methylation by the plants themselves [121]. The final product of the methylation pathway is the gas trimethyl arsine (TMAs (III)), which can be volatilized from the plant [78].

### 3.4. Disposal of Waste

**Figure 2:**
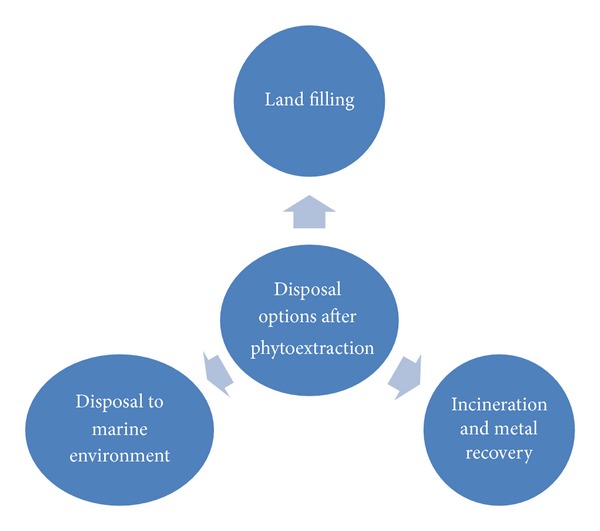
Various options for arsenic containing wastes disposal.

The contaminated plant biomass can be digested or ashed to reduce its volume (95%), and the resulting small volume of material can be processed as an “ore” to recover the contaminant (e.g., valuable heavy metals and radionuclides). If recycling the metal is not economically feasible, the relatively small amount of ash (compared to the original biomass or the extremely large volume of contaminated soil) can be disposed of in an appropriate manner [51]. Various disposal options have been presented in Figure 2.

Marine systems also have a particular ability to biotransform and detoxify inorganic arsenic, presumably due to their evolution in an arsenic-containing environment; seawater contains approximately 1 *μ*g As L^−1^ primarily as arsenate [130]. Arsenite added to seawater is likely to be quickly converted to arsenate, the more thermodynamically stable form [131]. The first stage of this detoxification process is formation of arsenosugars by algae; perhaps other organisms may also be involved and the final arsenic metabolite appears to be arsenobetaine, a stable nontoxic form of arsenic found in all marine animals [130]. Some marine unicellular algae can carry out this biotransformation at arsenate concentrations 1000-fold ambient levels (i.e. at 1000 *μ*g As L^−1^) [132]. Thus, it may be possible to dispose of the high arsenic fern directly into the open sea where it would degrade (contributing marginally to nutrient levels) and release inorganic arsenic which could be converted to nontoxic forms by natural processes. There may be possible ecological effects from the initial increased arsenic concentrations in seawater such as species changes in algae populations [133, 134].

## 4. Conclusions

Arsenic toxicity in soil and water is an increasing menace across the world and it is causing significant health damage to people living in developing and third world countries. It can be declared as a global hazard. Such a situation demands low-cost, technologically simple and point of use solutions to arsenic toxicity.

Phytoremediation is a sustainable option for developing countries which are hit by economic crisis and thus cannot afford technologically sophisticated solutions for their huge populations. Many plant species especially aquatic macrophytes and some wetland plants have shown promising ability to uptake arsenic from contaminated environments. Free metal ions in the soil solution are absorbed by plants and are reduced as metal chelates using specific high-affinity ligands (like oxygen-donor ligands, sulfur-donor ligands, and nitrogen-donor ligands). Bioaccumulation in stems and leaves along phytovolatilization have been shown to be possible tolerance mechanisms by plants against arsenic contamination.
