# Corrigendum to “Plants as Useful Vectors to Reduce Environmental Toxic Arsenic Content”

**DOI:** 10.1155/2018/7968934

**Published:** 2018-09-27

**Authors:** Nosheen Mirza, Qaisar Mahmood, Mohammad Maroof Shah, Arshid Pervez, Sikander Sultan

**Affiliations:** ^1^Department of Environmental Sciences, COMSATS Institute of Information Technology, Abbottabad 22060, Pakistan; ^2^Department of Microbiology and Molecular Genetics, University of the Punjab, Quaid-i-Azam Campus, Lahore 54590, Pakistan

The article titled “Plants as Useful Vectors to Reduce Environmental Toxic Arsenic Content” [1] was found to contain materials from Hinchman et al. [2] in Section 3.2, “Detoxification Mechanisms in Plants”, and Section 3.4, “Disposal of Waste”, which should have been cited.
